# P03-18 A Review of Sporting Role Model Led Interventions Promoting Physical Activity in Youth

**DOI:** 10.1093/eurpub/ckac095.054

**Published:** 2022-08-29

**Authors:** Eimear Kelly, Aoife Lane, Katie Liston, Kieran Dowd

**Affiliations:** Technological University of the Shannon: Midlands Midwest, Ireland

**Keywords:** Sporting Role Models, Physical Activity, Youth

## Abstract

**Background:**

Among adolescents there is a notable decline in physical activity (PA) levels from childhood, more notable among females than males. Visibility is regularly a cornerstone of strategy to promote engagement with women's sport particularly around stimulating and sustaining female participation in sport and PA. This includes leveraging sporting role models (SRM) to develop participation in sport and PA. The purpose of this research is to locate and describe the features and impact of SRM led interventions on female participation in sport and PA.

**Methods:**

APA PsychInfo, PudMed, and Sport Discus databases was used to identify SRM led interventions. In sum, 7,169 papers were identified with 360 duplicates. A further 5,122 were removed from title screen, 1,666 from abstract screening, 21 from full text review with two studies included in the final review.

**Results:**

Two SRM led interventions were included in the final review and were deemed as ‘fair' quality using Black and Downs Checklist. The first study1 aimed to improve attendance in PE, behaviour and attitude towards PA through a 12-week school based intervention. Schools were funded to complete a novel sport rolled out by teachers (group 1), with a second group having an additional visit from an elite ‘athlete mentor' (group 2). The study found 98% of students enjoyed the athlete visits and found them beneficial however there was no additional impact on PE attendance and attitudes to PA linked to athlete visits. The second study2 involved school students visiting a local professional football club and meeting professional football players through video messages and letters over a four-month school period. The intervention group showed a significant increase in self-efficacy towards PA compared to the control group.

**Conclusion:**

There is limited, if any, evidence to support SRM led interventions to promote participation in sport or PA. Studies considered in this review are poorly designed and evaluated and lack theoretical frameworks. There is scope to undertake a broader review of grey literature due to the scarcity of published evidence despite anecdotal support and implementation of SRM interventions throughout the sport sector.

